# Improved glycaemia during the Covid-19 pandemic lockdown is sustained post-lockdown and during the “Eat Out to Help Out” Government Scheme, in adults with Type 1 diabetes in the United Kingdom

**DOI:** 10.1371/journal.pone.0254951

**Published:** 2021-07-20

**Authors:** Parizad Avari, Rebecca Unsworth, Siân Rilstone, Chukwuma Uduku, Karen M. Logan, Neil E. Hill, Ian F. Godsland, Monika Reddy, Nick Oliver

**Affiliations:** 1 Department of Metabolism, Digestion and Reproduction, Imperial College London, London, United Kingdom; 2 Department of Paediatrics, St Mary’s Hospital, Imperial College Healthcare NHS Trust, London, United Kingdom; Medizinische Universitat Graz, AUSTRIA

## Abstract

**Aims:**

The majority of studies report that the Covid-19 pandemic lockdown did not have a detrimental effect on glycaemia. We sought to explore the impact of lockdown on glycaemia and whether this is sustained following easing of restrictions.

**Methods:**

Retrospective, observational analysis in adults and children with type 1 diabetes attending a UK specialist centre, using real-time or intermittently scanned continuous glucose monitoring. Data from the following 28-day time periods were collected: (i) pre-lockdown; (ii) during lockdown; (iii) immediately after lockdown; and (iv) a month following relaxation of restrictions (coinciding with Government-subsidised restaurant food). Data were analysed for times in glycaemic ranges and are expressed as median (IQR).

**Results:**

145 adults aged 35.5 (25.8–51.3) years with diabetes duration of 19.0 (7.0–29.0) years on multiple daily injections of insulin (60%) and continuous insulin infusion (40%) were included. In adults, % time in range (70-180mg/dL) increased during lockdown (60.2 (45.2–69.3)%) compared to pre-lockdown (56.7 (43.5–65.3)%; p<0.001). This was maintained in the post-lockdown time periods. Similarly, % time above range (>180mg/dL) reduced in lockdown compared to pre-lockdown (p = 0.01), which was sustained thereafter. In children, no significant changes to glycaemia were observed during lockdown. In multivariable analysis, a greater increase in %TIR 3.9-10mmol/L (70-180mg/dL) during lockdown was associated with higher levels of deprivation (coefficient: 4.208, 95% CI 0.588 to 7.828; p = 0.02).

**Conclusions:**

Glycaemia in adults improved during lockdown, with people from more deprived areas most likely to benefit. This effect was sustained after easing of restrictions, with government-subsidised restaurant eating having no adverse impact on glycaemia.

## 1.0 Introduction

The coronavirus disease (COVID-19) outbreak caused by the novel Severe Acute Respiratory Syndrome Coronavirus 2 (SARS-CoV-2) was defined by the World Health Organisation as a global pandemic on 11^th^ March 2020 [1]. The UK government imposed a strict lockdown to slow the transmission of SARS-CoV-2 on 23^rd^ March 2020 [2]. Both type 1 and type 2 diabetes are associated with increased mortality in hospitalized patients with COVID-19 [3], with a higher number of deaths in people with type 1 and type 2 diabetes living in the most deprived neighbourhoods compared to those living in the least deprived areas [3]. Furthermore, among people with diabetes, factors such as hyperglycaemia and obesity are independently associated with COVID-19 mortality [4]. SARS-CoV-2 may adversely affect glucose metabolism both directly by causing beta cell death and indirectly by increasing insulin resistance [5].

Type 1 diabetes (T1D) self-management can be challenging, involving multiple daily injections of insulin (MDI) or continuous subcutaneous insulin infusion (CSII), guided by self-monitoring of glucose [6].

Previous studies have compared glycaemic measures before and during lockdown in people with T1D using different glucose monitoring and insulin delivery modalities with the majority finding that lockdown has not had a detrimental effect [7–13]. Few studies have investigated glycaemic metrics following the end of lockdown during the relaxation of restrictions. These studies demonstrated sustained improvements for up to a 2-week period but have not investigated further [14–17].

In the UK, following lockdown, one of the Government’s policy measures to support businesses reopening, was the “Eat Out to Help Out Scheme (EOHO)”. Throughout August 2020, the EOHO scheme supported the hospitality sector by providing consumers a discount of up to 50% off food and drink (non-alcoholic) consumed in participating outlets with a maximum discount of £10 per meal per person, valid all day Monday, Tuesday and Wednesday. These included restaurants and pubs, with > 78,000 outlets participating and over 160 million meals subsidised [18].

We aimed to explore the impact of lockdown and during the relaxation of restrictions in a diverse group of adults and children with T1D living in London and to assess the factors impacting glycaemia during this unprecedented change to people’s lifestyles, including use of glucose monitoring technologies or CSII and pre-lockdown glycaemic control (HbA1c and time in acceptable glycaemia range). We also explored the influence of deprivation according to the English indices of deprivation [19].

## 2.0 Methods

### 2.1 Inclusion and exclusion criteria

This is a retrospective observational analysis of all adults and children with T1D using real-time continuous glucose monitoring (rtCGM; Dexcom G6 or Medtronic) or intermittently scanned continuous glucose monitoring (isCGM; Freestyle Libre), who were under regular care at Imperial College Healthcare NHS Trust (ICHNT) in London, UK. The groups were defined by the responsible clinical service as paediatric (<18 years old) or adult (≥18 years old). ICHNT is a specialist diabetes referral centre for northwest London, providing services to a diverse urban population. In addition, anonymised data from participants using rtCGM as part of the free-living research study: ’*Assessment of the Impact of Real-Time Continuous Glucose Monitoring on People Presenting With Severe Hypoglycaemia (AIR-CGM; NCT03748433)*’ [20] at Imperial College London, were included.

Only participants with at least 70% of continuous glucose data available for both 28 day periods pre- and during lockdown were included. The requirement for at least 70% of glycaemic data was based on consensus recommendations for reporting % times in range [21]. Participants who did not meet the above criteria, had type 2 diabetes or women who were pregnant were excluded from analysis. The sample in these analyses may be considered representative of a larger population, however only reflects individuals with T1D on rtCGM or isCGM, and not those self-monitoring blood glucose.

### 2.2 Methods

#### 2.2.1 Data collection and computation of glycaemic outcomes

Continuous glucose data were collected over 28 days prior to lockdown (15th February 2020 00:00hrs to 13th March 2020 23:59hrs) and compared with data of the same duration during lockdown (24th March 2020 00:00hrs to 20th April 2020 23:59hrs), immediately after lockdown (4^th^ July 2020 00:00hrs to 31^st^ July 2020 23:59hrs); and a month following relaxation of restrictions (1^st^ August 2020 00:00hrs to 28^th^ August 2020 23:59hrs) coinciding with the EOHO Government scheme in the U.K.

The study was entirely observational (with no deviation from standard clinical care) and ethics approval was not required. All individuals granted specific permission to share their glucose data with the clinical teams, and for clinic staff to access this, when linking their devices and uploading their data to web-based online software (Dexcom Clarity, LibreView, and Tidepool). NHS Research Ethics Committee review was not required. Data for analysis were de-identified before they were analysed, with participants identified solely by study number.

Percentage (%) times in glycaemic range and measures of glycaemic variability (GV) were computed using EasyGV (v10.0) software [22]. Outcomes measures included % time in range (TIR) 3.9–10mmol/L (70 -140mg/dL), % time above range >10mmol/L (>180mg/dL; TAR1) and >13.9mmol/L (>250mg/dL; TAR2), and % time below range (TBR) <3.9mmol/L (<70mg/dL; TBR1), <3.0mmol/L (<54mg/dL; TBR2), and <2.8mmol/L (<50mg/dL; TBR3). Evaluated GV measures include standard deviation (SD), coefficient of variation (CV), low blood glucose index (LBGI) and mean absolute glucose change per unit time (MAG). Estimated glucose management index (GMI) was calculated using the following formula [23]: GMI (mmol/mol) = 12.71 + 4.70587 x [mean glucose (mmol/L)].

There were no available data on lifestyle or therapy changes for diet, exercise or insulin use. IsCGM scanning frequency during these timeframes has been included in the analysis.

Socioeconomic deprivation was assessed by the English Indices of Deprivation 2019 [19] using postcodes. Deprivation deciles are based on the Index of Multiple Deprivation 2019 (IMD 2019). Decile 1 represents the most deprived 10% of neighbourhoods in England, whilst decile 10 represents the least deprived 10%.

#### 2.2.2 Statistical methods

Changes in glucose outcome measures were analysed between baseline (pre-lockdown), during lockdown and for the two months post lockdown. Data (%TIR) were tested for normality using the Shapiro–Wilk test (p<0.05). The adult and paediatric groups were also analysed separately. Differences between pre- and during lockdown glucose outcomes measures were tested for significance using Wilcoxon matched-pairs signed-rank tests. Sub-analysis comparisons in the adult cohort, justified by relatively large numbers of observations and significant variation between time points, explored differences according to insulin delivery and mode of glucose monitoring.

Further analysis was performed in those participants who also had at least 70% of glucose data for the two periods after lockdown. The non-parametric repeated measures Friedman statistical test was used to detect differences in glycaemia across the four different time points. To determine where the significant differences lay between the four time points for the adult cohort, the Wilcoxon matched-pairs signed-rank tests were performed as pairwise analyses.

Multivariable linear regression analysis of variation in glycaemic outcome measures was performed using the covariates: age, gender, sensor modality, insulin delivery method, baseline %TIR, baseline HbA1c and deprivation. Deprivation deciles were re-categorised according to the tertiles: severely deprived, moderately deprived and least deprived, the equal numbers in each category then providing for equal category weightings in the regression model. This analysis derived from observational data acquired in an unprecedented situation. Accordingly, no information was available for a prior power calculation and a threshold of statistical significance of p<0.05 was adopted as an aid to interpretation rather than as a decision-making criterion. Statistical analyses were performed using Stata version 13 (StataCorp, College Station, TX).

## 3.0 Results

### 3.1 Baseline demographics

We identified 145 individuals with at least 70% data uploaded pre- and during lockdown (S1 Fig). For adults and paediatrics combined, the median (interquartile range) age was 35.5 (25.8–51.3) years with diabetes duration of 19.0 (7.0–29.0) years and HbA1c of 7.7 (7.2–8.6)% (61 (55–70) mmol/mol). 75 participants (52%) were male, 58 (40%) were CSII users and 48 (33%) used rtCGM; 20 (14%) used both rtCGM and CSII. For the adults using rtCGM, more than a quarter (28%) had impaired awareness of hypoglycaemia (GOLD score ≥ 4) and/or ≥ 1 episode of severe hypoglycaemia in the past 1 year. Baseline characteristics of the participants are summarized for adults and paediatrics in Table 1.

**Table 1:** Baseline demographics for the complete dataset and separate adult and paediatric cohorts.

Demographics	Combined (n = 145)	Adults (n = 121)	Paediatrics (n = 24)
Gender (male)	75 (52%)	59 (49%)	16 (59%)
Age (years)	35.5 (25.8–51.3)	40.0 (31.4–53.1)	13.1 (9.5–14.5)
Duration of diabetes (years)	19.0 (7.0–29.0)	22.0 (12.0–31.8)	4.7 (2.1–7.0)
Insulin delivery modality			
CSII users	58 (40%)	43 (36%)	15 (63%)
MDI users	87 (60%)	78 (64%)	9 (38%)
Glucose sensing modality			
rtCGM users	48 (33%)	43 (36%)	5 (21%)
isCGM users	97 (67%)	78 (64%)	19 (79%)
HbA1c (%)	7.7 (7.2–8.6)	7.6 (7.1–8.5)	7.9 (7.4–8.9)
HbA1c (mmol/mol)	61 (55–70)	60 (54–69)	63 (57–74)
Deprivation Index Score	4.0 (3.0–7.0)	4.0 (3.0–7.0)	4.0 (2.0–5.0)

Results are expressed as median (IQR)/ n (%).

### 3.2 Improved glycaemia during lockdown in adults

For adults during lockdown, %TIR increased from 56.7 (43.5–65.3)% to 60.2 (45.2–69.3)% (Table 2; p <0.001). A similar change was observed for % time in euglycaemia 3.9–7.8mmol/L (70-140mg/dL; Table 2; p = 0.001). In the sub-analysis by insulin delivery (CSII vs MDI; S1 Table) and glucose sensing modality (rtCGM vs isCGM; S2 Table), significant improvements in %TIR were observed for all subgroups. The median change in %TIR difference between pre- and during lockdown was +2.1(-1.1 to +5.6)% in the MDI group (p = 0.004); +3.1(-3.3 to +7.2)% in the CSII group (p = 0.05); +3.3 (-2.9 to +5.6)% in the rtCGM group (p = 0.04) and +2.4 (-1.8 to +6.9)% in the isCGM group (p = 0.005). There were no significant differences for median change in %TIR between the subgroups based on insulin delivery (MDI vs CSII) nor glucose sensing modality (isCGM vs rtCGM).

**Table 2:** Pairwise analysis of glycaemic outcomes in the adult and paediatric cohorts.

	Adults			Paediatrics		
	Pre-lockdown (n = 121)	During lockdown (n = 121)	P-value (Pre- vs During lockdown)	Pre-lockdown (n = 24)	During lockdown (n = 24)	P-value (Pre- vs During lockdown)
**% time in range**						
TIR: 3.9-10mmol/L (70-180mg/dL)	56.7 (43.5–65.3)	60.2 (45.2–69.3)	<0.001*	45.4 (33.8–60.8)	50.8 (35.0–61.1)	0.24
**% time in euglycaemia**						
3.9–7.8mmol/L (70-140mg/dL)	33.7 (24.5–39.9)	36.0 (25.6–45.6)	0.001*	28.3 (20.1–36.1)	32.2 (21.4–41.0)	0.35
**% time in hypoglycaemia**						
TBR1: <3.9mmol/L (<70mg/dL)	3.8 (1.5–7.3)	3.8 (1.5–7.0)	0.38	3.2 (1.0–6.6)	3.1 (1.1–5.4)	0.86
TBR2: <3.0mmol/L (<54mg/dL)	0.7 (0.2–1.9)	0.9 (0.3–2.1)	0.25	0.6 (0.1–1.3)	0.5 (0.2–1.6)	0.93
TBR3: <2.8mmol/L (<50mg/dL)	0.5 (0.1–1.4)	0.5 (0.1–1.5)	0.10	0.3 (0.0–0.7)	0.3 (0.1–1.1)	0.95
**% time in hyperglycaemia**						
TAR1: >10mmol/L (>180mg/dL)	38.2 (27.6–52.4)	34.1 (24.0–48.8)	0.01*	43.2 (37.0–61.6)	39.7 (33.5–62.1)	0.57
TAR2: >13.9mmol/L (>250mg/dL)	11.5 (5.9–18.3)	9.2 (4.6–17.3)	0.007*	20.7 (9.1–34.8)	18.5 (9.9–31.9)	0.35
**Glycaemic variability measures**						
Mean	9.2 (8.4–10.2)	9.0 (8.0–10.1)	0.02*	9.9 (9.1–12.1)	9.6 (8.8–11.7)	0.51
GMI (%)	7.3 (6.9–7.7)	7.2 (6.8–7.7)	0.02*	7.6 (7.2–8.5)	7.5 (7.1–8.4)	0.51
GMI (mmol/L)	56.0 (52.2–60.7)	55.1 (50.8–60.2)	-	59.2 (55.4–69.8)	58.0 (54.2–67.8)	-
Standard deviation	3.6 (3.0–4.1)	3.4 (2.9–3.9)	<0.001*	4.4 (3.8–4.9)	4.1 (3.6–4.7)	0.07
CV (%)	37.6 (34.5–42.8)	37.4 (33.3–41.0)	0.008*	41.2 (38.0–47.0)	41.6 (36.9–44.7)	0.25
LBGI	1.0 (0.5–1.8)	1.0 (0.5–1.7)	0.49	0.9 (0.4–1.5)	0.9 (0.4–1.3)	0.63
MAG	2.5 (2.2–3.0)	2.5 (2.1–2.8)	<0.001*	3.2 (2.8–3.4)	3.0 (2.5–3.3)	0.01*

Participants used rtCGM/ isCGM for at least 70% of time with at least 70% data uploaded for both time periods (as per consensus recommendations [21]). Results are expressed as median (IQR).

*p<0.05. Abbreviations: CV, coefficient of variation; GMI, glucose management indicator; LBGI, low blood glucose index; MAG, mean absolute glucose; TAR, time above range; TBR, time below range; TIR, time in range.

For %TAR1 and %TAR2, significant reductions were observed during the lockdown period (p = 0.01 and p = 0.007 respectively; Table 2). In the sub-analyses, a significant reduction was seen in %TAR1 for rtCGM users (median change -3.0 (-8.0 to +1.2)%; p = 0.01); and in %TAR2 for isCGM users (median change -0.9 (-3.4 to +0.9)%; p = 0.02). There were no between-group differences for MDI vs CSII, nor isCGM vs rtCGM.

For hypoglycaemia %TBR1 and %TBR2, no significant difference was observed from baseline to lockdown (Table 2). In the MDI group, significant reductions in hypoglycaemia for %TBR1 and %TBR3 were observed (p = 0.02 and p = 0.04 respectively). Between group differences revealed significant reduction in %TBR1 in MDI participants compared to CSII (p = 0.005). Significantly reduced hypoglycaemia was also noted in the group using isCGM for all %TBR thresholds, with no significant change in hypoglycaemia in the rtCGM users. There were no between-group differences in hypoglycaemia for the isCGM and rtCGM groups.

Mean glucose values and measures of variability (SD, CV and MAG) significantly reduced during lockdown in adults, except LBGI (p<0.05; Table 2). Although trends were similar across all cohorts, significance was reached predominantly in MDI and isCGM users.

For individuals using isCGM, there were no significant differences in the number of independent scans performed by each individual (pre-lockdown 11 (7–16) scans/day vs during lockdown 10.5 (7–15) scans/day; p = 0.18).

### 3.3 Glycaemic outcomes in paediatrics

In the paediatric analysis pre- and during lockdown %TIR was 45.4 (33.8–60.8)% and 50.8 (35.0–61.1)% respectively, % time in euglycaemia was 28.3 (20.1–36.1)% and 32.2 (21.4–41.0)% respectively, with no statistically significant differences (Table 2). The %TBR did not differ before and during lockdown (Table 2). The changes in glycaemic variability measures are summarised in Table 2, with no statistically significant differences, except for a reduction in MAG from 3.2 (2.8–3.4) pre-lockdown to 3.0 (2.5–3.3) during lockdown (p = 0.01).

Similar to adults, there were no significant differences in the number of independent scans performed in the paediatric cohort.

### 3.4 Independent influences on lockdown glycaemia

Multivariable linear regression analysis (Table 3) suggested greater social deprivation was associated with an increase in %TIR (coefficient: 4.208 [95% CI 0.588 to 7.828]; p = 0.02) with a negative association with change in %TAR1 (coefficient: -4.746 [95% CI -8.771 to -0.721]; p = 0.02).

**Table 3:** Multiple linear regression analysis of predictors of change (Δ) in %times in range and CV for blood glucose from pre-lockdown to lockdown for the combined adult and paediatric cohorts (n = 145).

	Δ%TIR 3.9-10mmol/L (70-180mg/dL)	Δ%TBR1 <3.9mmol/L (<70mg/dL)	Δ%TBR2 <3.0mmol/L (<54mg/dL)	Δ%TAR1 >10mmol/L (>180mg/dL)	ΔCV(%)
**Age**	0.029 (-0.061, 0.119)^0.5^	0.015 (-0.017, 0.047)^0.3^	0.006 (-0.009, 0.022)^0.4^	-0.044 (-0.144, 0.056)^0.4^	0.000 (-0.000, 0.000)^0.8^
**Men**	0.861 (-2.047, 3.769)^0.6^	-0.795 (-1.824, 0.233)^0.1^	-0.269 (-0.784, 0.247)^0.2^	-0.071 (-3.304, 3.163)^1.0^	-0.009 (-0.023, 0.006)^0.2^
**CGM use**	0.128 (-2.997, 3.252)^0.9^	1.498 (0.393, 2.603)^**0.008**^	0.518 (-0.036, 1.072)^0.1^	-1.586 (-5.060, 1.888)^0.4^	0.019 (0.004, 0.035)^**0.01**^
**CSII use**	2.361 (-0.689, 5.410)^0.1^	0.991 (-0.087, 2.070)^0.1^	0.362 (-0.178, 0.903)^0.2^	-3.332 (-6.722, 0.059)^**0.05**^	0.012 (-0.003, 0.027)^0.1^
**Pre-lockdown HbA1c**	-0.094 (-0.254, 0.066)^0.2^	-0.045 (-0.102, 0.011)^0.1^	-0.008 (-0.036, 0.021)^0.6^	0.136 (-0.042, 0.314)^0.1^	0.000 (-0.001, 0.001)^1.0^
**Pre-lockdown TIR**	-0.560 (-0.186, 0.066)^0.4^	-0.023 (-0.067, 0.022)^0.3^	0.003 (-0.020, 0.025)^0.2^	0.082 (-0.058, 0.222)^0.2^	0.000 (-0.000, 0.001)^0.4^
**Deprivation**					
Moderate (IMD deciles 4–5)	2.884 (-0.792, 6.560)^0.1^	0.723 (-0.577, 2.023)^0.3^	0.350 (-0.302, 1.002)^0.3^	-3.472 (-7.559, 0.616)^0.1^	0.016 (-0.002, 0.035)^0.1^
Severe (IMD deciles 1–3)	4.208 (0.588, 7.828)^**0.02**^	0.615 (-0.665, 1.895)^0.3^	-0.022 (-0.664, 0.620)^0.9^	-4.746 (-8.771, -0.721)^**0.02**^	0.007 (-0.011, 0.254)^0.4^

Coefficients (95% confidence intervals) are shown and significances in superscript. Abbreviations: CGM, continuous glucose monitoring; CSII, continuous subcutaneous insulin infusion; CV, coefficient of variation; IMD, index of multiple deprivation; TAR, time above range; TBR, time below range; TIR, time in range.

There was a significant association between rtCGM use and an increase in %TBR1 during lockdown (coefficient 1.498, 95% CI 0.393 to 2.603; p = 0.008). This association was not observed with %TBR2 (coefficient: 0.518, 95% CI -0.036 to 1.072; p = 0.10). Increased %CV was also associated with rtCGM use during lockdown (coefficient: 0.019, 95% CI 0.004 to 0.035; p = 0.01). Age, gender, baseline HbA1c, baseline %TIR and insulin delivery method did not influence the effect of lockdown on %TIR.

### 3.5 Effect of deprivation

The overall median (IQR) deprivation deciles across the cohort for adults and children were 4 (3–7) (Table 1). At baseline, there were no significant differences in HbA1c between the two groups (62 (55–73) mmol/mol in deciles ≤ 4 vs 59 (55–67) mmol/mol in deciles ≥ 5; p = 0.12).

Individuals in the lower 50% of the deprivation scores (deciles ≤ 4 vs deciles ≥ 5) had significantly greater change in %TIR during lockdown than those in the upper 50% (change %TIR +3.25 (-0.38 to 8.71) vs +1.79 (-3.61 to 4.73) respectively; p = 0.02). The boxplot (S2 Fig) illustrates the change in %TIR for adults and children categorised into tertiles of index of deprivation.

### 3.6 Post-lockdown analysis

Further analysis was performed in the adult cohort who had data uploaded in the two months following the lockdown (the period 1 month after lockdown coinciding with the EOHO government scheme) (Table 4); n = 97 (S1 Fig). Between during and post-lockdown periods, there were no significant differences in %TIR. Similarly, the significant reduction in %TAR1 observed in lockdown compared to pre-lockdown (p = 0.04), was sustained thereafter. %TBR1 did not change throughout the 4 time periods.

**Table 4:** Glycaemic outcomes in the pre-, during, immediately after and a month after lockdown (n = 97).

	Adults (n = 97)				
	Pre-lockdown	During lockdown	Immediately after lockdown	1 month after lockdown	p-value
**% time in range**					
TIR: 3.9-10mmol/L (70 -180mg/dL)	56.7 (43.5–65.1)	60.2 (43.9–69.3)	59.5 (48.6–71.5)	59.1 (46.7–72.2)	<0.001*^†‡^
**% time in euglycaemia**	34.9 (24.5–39.9)	36.1 (24.9–45.7)	35.1 (26.1–44.3)	34.6 (25.4–47.4)	0.010*^†‡^
3.9–7.8mmol/L (70 -140mg/dL)					
**% time in hypoglycaemia**					
TBR1: <3.9mmol/L (<70mg/dL)	3.8 (1.5–6.6)	3.8 (1.5–6.5)	3.7 (1.4–7.0)	3.4 (1.4–6.6)	0.59
TBR2: <3.0mmol/L (<54 mg/dL)	0.8 (0.3–1.7)	0.9 (0.2–2.0)	0.8 (0.1–1.8)	0.6 (0.2–1.9)	0.55
TBR3: <2.8mmol/L (<50mg/dL)	0.5 (0.1–1.1)	0.5 (0.1–1.3)	0.5 (0.0–1.2)	0.3 (0.1–1.3)	0.64
**% time in hyperglycaemia**					
TAR1: >10mmol/L (>180mg/dL)	38.0 (27.6–49.0)	33.8 (23.6–50.8)	34.9 (24.0–47.2)	36.4 (21.3–49.9)	<0.001*^†‡^
TAR2: >13.9 mmol/L (>250mg/dL)	11.3 (5.9–18.3)	8.4 (4.4–17.4)	8.8 (3.3–17.0)	8.8 (2.6–17.1)	<0.001*^†‡^
**Glycaemic variability measures**					
Mean	9.2 (8.4–10.2)	9.0 (8.0–10.1)	9.1 (8.1–10.4)	9.1 (7.8–10.3)	0.004*^†‡^
GMI (%)	7.3 (6.9–7.7)	7.2 (6.8–7.7)	7.2 (6.8–7.8)	7.2 (6.7–7.7)	0.004*^†‡^
GMI (mmol/mol)	55.9 (52.0–60.8)	54.9 (50.3–60.4)	55.3 (50.8–61.6)	55.6 (49.5–61.0)	<0.001*^†‡^
Standard deviation	3.6 (3.0–4.1)	3.4 (2.8–3.8)	3.4 (2.8–3.9)	3.4 (2.8–3.8)	0.002*^‡^
CV (%)	38.8 (34.1–41.8)	37.6 (33.5–40.8)	37.3 (33.2–40.9)	36.5 (32.9–40.2)	0.75
LBGI	0.9 (0.5–1.6)	1.0 (0.5–1.6)	0.9 (0.4–1.9)	0.9 (0.4–1.7)	<0.001 *^†‡^
MAG	2.5 (2.2–3.0)	2.4 (2.1–2.8)	2.0 (1.0–2.3)	1.9 (1.0–2.3)	<0.001*^†‡^

Results expressed as median (IQR). P-value calculated by Friedman test comparing glycaemic variables between the four time periods. For between group sub-analysis

*pre-lockdown vs during lockdown p<0.05analysis

†pre-lockdown vs immediately after lockdown p<0.05analysis

‡ pre-lockdown vs 1 month after lockdown p<0.05. Abbreviations: CV, coefficient of variation; GMI, glucose management indicator; LBGI, low blood glucose index; MAG, mean absolute glucose; TAR, time above range; TBR, time below range; TIR, time in range.

In the paediatric cohort, thirteen children also had at least 70% data uploaded in the two periods after lockdown (S1 Fig). There was no statistically significant difference across the four time periods in %TIR, %TAR1 and %TAR2, or %TBR1 (S3 Table). %TBR3 demonstrated significant variation (p = 0.03) with median percentages during the four observation periods of 0.08, 0.24, 0.18 and 0.05, respectively.

## 4.0 Discussion

These real-world data from a large specialist centre suggest that adults in an urban setting with T1D using glucose sensing technologies had an increase in time in range, with an associated reduction in time above range and reductions in measures of glucose variability during lockdown. Exposure to hypoglycaemia and hypoglycaemia risk assessed by LBGI did not change. Improvement in time in range was associated with being in the most deprived tertile, as was the reduction in time above range. This study adds to a growing body of evidence suggesting that lockdown is not associated with a deleterious effect on measures of glycaemia [7–10, 13–16, 24, 25].

The improved glycaemia in the adult cohort during this period of lockdown is likely to reflect the complex interplay of social, behavioural and environmental factors previously reported [7, 25, 26]. Even studies demonstrating a higher food intake [7] or specifically increased carbohydrate consumption without an increase in total daily insulin dose [16], more frequent snacking during lockdown [7], reduced physical activity [7, 25] and weight gain [25], did not find that lockdown had a detrimental effect on glycaemia. Perhaps the impact of these factors was outweighed by the imposed changes such as working from home, allowing for more frequent checking blood glucose values and meal eating habits, which are associated with better glycaemia control [27]. Meal eating habits are associated with daily routine [27], and people with diabetes have reported more regular mealtimes during lockdown [7]. Although individuals had altered access to usual diabetes care with reduced face-to-face contact during lockdown, remote support was provided by telephone or e-mail and this may have been more accessible for some people who find it challenging to attend clinics. A Spanish survey found that 97.9% agreed with the use of telemedicine, favouring telephone as their preferred means of communication [26].

Our study did not demonstrate an increase in scanning frequency in isCGM users. However, this only made up 64% of the adult cohort. In addition, despite greater scanning frequency, how individuals use that data is critical, and imposed restrictions on working from home may allow for improved lifestyle choices.

Other studies investigating the two week period following the end of lockdown, have found comparable results to our study, showing a sustained increase in %TIR and reduction in %TAR1 [14, 15] or TAR 251-400mg/dL [16]. A small Spanish dataset of participants on MDI using isCGM, found no change in glycaemic metrics between pre and during lockdown but an improvement in %TIR (p = 0.05) and %TAR1 and %TAR2 post lockdown compared to before [28].

The numbers in the paediatric cohort were small and changes did not reach statistical significance, but effect sizes were similar to those seen adults. Our results are comparable to other studies in children and adolescents which reported no significant increase in %TIR during lockdown [11, 29, 30]. Conversely, other studies have demonstrated an increase in %TIR for participants [12], including those using hybrid closed loop systems [10, 13]. Reports in children have shown reduced time for physical activities [12], an increase in screen time [31], an increase in consumption of sweet and fried food in adolescents [32] and a change to meal patterns [30], emphasising the complex social factors involved. Nevertheless, many of the factors contributing to improved glycaemia in adults are likely to be shared in children. In addition, increased parental supervision during lockdown may have influenced behaviour, including the frequency and timing of insulin delivery in relation to food, particularly considering that 25 percent of adolescents report missed insulin injections [33]. The significant reduction in MAG in paediatrics during lockdown suggests decreased glucose variation which may in part reflect better insulin matching to food, given inaccurate carbohydrate counting has been associated with higher glycaemic variability in adults [34].

A small Italian dataset in adolescents during partial relaxation of restrictions and at the end of lockdown, found a sustained improvement in %TIR and a reduction in %TBR from lockdown onwards compared to pre lockdown [17]. However, all these participants used hybrid closed loop system with an increase in percentage time in auto mode at the end of lockdown compared to pre lockdown [17].

Multivariable analysis in adults and paediatrics indicated people in the lower 50% of the deprivation scores improved %TIR more than those in the upper 50%. The cause for this novel finding is unclear, but, with lower dietary quality scores in the most disadvantaged [35], may reflect reduced access to less healthy lifestyle choices, increased time and capacity for self-management tasks, or increased engagement with education and support as remote consultations may be less burdensome and more accessible than clinic visits. Furthermore, lower socioeconomic status is associated with higher HbA1c as seen in the T1D Exchange registry, which may enable greater room for changes in glycaemia towards improvement [36]. Our results differ from the findings in Scotland, which suggested higher levels of socioeconomic deprivation were an independent predictor of deteriorating glycaemic control [8].

In our regression model, rtCGM use was likely to be associated with increased %TBR1 hypoglycaemia during lockdown despite no overall increase in time below range being observed in between-group comparisons. This association was not observed for %TBR2. In the UK, rtCGM is reimbursed by the National Health Service (NHS) for people with T1D at highest risk of hypoglycaemia, including those experiencing severe hypoglycaemia, loss of awareness of hypoglycaemia, hypoglycaemia that is causing problems with daily activities, and extreme fear of hypoglycaemia [37]. Behaviours associated with exposure to hypoglycaemia [38] may be enabled during lockdown, increasing exposure to mild hypoglycaemia, but the absence of an association with clinically important hypoglycaemia is reassuring. A reduction was seen in hypoglycaemia in people using isCGM but not rtCGM. This may reflect the lower TBR in the rtCGM group at baseline as the addition of alerts and alarms, even in a higher risk group, kept hypoglycaemia to a minimum and further improvement was not detectable.

The strengths of this study include inclusion of adults and children, inclusion of people at highest risk of hypoglycaemia and a large, diverse population of people using CSII and MDI regimens. We analysed study periods of 28 days, increasing the robustness of the CGM derived metrics, with a longer time period analysed than other studies pre and during lockdown [7–9, 39, 40] and also after lockdown [14–17, 28]. Although >70% of 14 days rtCGM data is the minimum recommended period for most evaluated metrics [21], metrics related to hypoglycaemia in particular require longer duration due to higher error and variance [41]. Very importantly, we include deprivation in our analysis.

Limitations to our analyses include missing insulin, dietary intake, education, occupation, and physical activity data. Also, further analyses additionally including people using capillary blood glucose testing would have value. In identifying likely prominent features of glycaemia during lockdown, we carried out a number of statistical tests and it is possible that some significances may have arisen by chance. Nevertheless, our principal outcome variables: %TIR and CV were both significant at p<0.01, and statistical testing was generally used as a guide to interpretation in our analysis. Our findings could be regarded as hypothesis-generating, but it should be emphasised that lockdown constituted an unprecedented change in lifestyle.

In conclusion, glycaemia in adults improved during lockdown, particularly in people from more deprived areas, and was sustained post relaxation of lockdown. The improved glycaemia is likely to be a result of complex social, behavioural, and environmental factors influencing lifestyle during COVID-19. A similar effect size for glycaemic outcomes was observed in children, but the smaller sample size limited definitive conclusions. There was no deterioration in glycaemia associated with lifestyle during the EOHO Government scheme in the UK. Future studies including a larger number of children are recommended, as are detailed analyses of the differential behaviour changes seen across deprivation categories.

## Supporting information

S1 Table Pairwise analysis of glycaemic outcomes for CSII and MDI users in adults. Participants used rtCGM/ isCGM for at least 70% of time with at least 70% data uploaded for both time periods (as per consensus recommendations). All data presented as median (IQR). Abbreviations: CSII, continuous subcutaneous insulin infusion; CV, coefficient of variation; GMI, glucose management indicator; LBGI, low blood glucose index; MAG, mean absolute glucose; MDI, multiple daily injections of insulin; TAR, time above range; TBR, time below range; TIR, time in range.
(DOCX)

S2 Table Pairwise analysis of glycaemic outcomes for rtCGM and isCGM users in adults. Participants used rtCGM/ isCGM for at least 70% of time with at least 70% data uploaded for both time periods (as per consensus recommendations). All data presented as median (IQR). Abbreviations: CV, coefficient of variation; GMI, glucose management indicator; isCGM, intermittently scanning continuous glucose monitoring; LBGI, low blood glucose index; MAG, mean absolute glucose; rtCGM, real-time continuous glucose monitoring; TAR, time above range; TBR, time below range; TIR, time in range.
(DOCX)

S3 Table Paediatric analysis of glycaemic outcomes pre-, during, immediately after and a month after lockdown (n = 13). Participants used rtCGM/ isCGM for at least 70% of time with at least 70% data uploaded for all four time periods (as per consensus recommendations). P-value calculated by Friedman test comparing glycaemic variables between the four time periods. All data presented as median (IQR). Abbreviations: CV, coefficient of variation; GMI, glucose management indicator; LBGI, low blood glucose index; MAG, mean absolute glucose; TAR, time above range; TBR, time below range; TIR, time in range.
(DOCX)

S1 Fig Recruitment flowchart. Abbreviations: FSL, flash glucose monitoring; rtCGM, real-time continuous glucose monitoring.
(DOCX)

S2 Fig Box plot to demonstrate the change in %TIR by tertiles for adults and children included in the pair-wise analysis (n = 145) using the English index of multiple deprivation 2019. The “most deprived” tertile include deciles 1–3 (n = 52), the “moderately deprived” tertile includes deciles 4–5 (n = 43) and the “least deprived” tertiles includes deciles 6–10 (n = 50). Abbreviations: TIR, time in range.
(DOCX)
