# Supplementary material for: Improved glycaemia during the Covid-19 pandemic lockdown is sustained post-lockdown and during the “Eat Out to Help Out” Government Scheme, in adults with Type 1 diabetes in the United Kingdom
Source: PLoS One. 2021 Jul 20;16(7):e0254951. doi: 10.1371/journal.pone.0254951 (PMC8291633; doi:10.1371/journal.pone.0254951)
Supplement: S1 Table — Participants used rtCGM/ isCGM for at least 70% of time with at least 70% data uploaded for both time periods (as per consensus recommendations). All data presented as median (IQR). Abbreviations: CSII, continuous subcutaneous insulin infusion; CV, coefficient of variation; GMI, glucose management indicator; LBGI, low blood glucose index; MAG, mean absolute glucose; MDI, multiple daily injections of insulin; TAR, time above range; TBR, time below range; TIR, time in range. (DOCX) [file pone.0254951.s001.docx]

|  | **CSII (n=43)** | | | **MDI (n=78)** | | |
| --- | --- | --- | --- | --- | --- | --- |
|  | **Pre-lockdown** | **During lockdown** | **P-value**  **(Pre- vs During Lockdown)** | **Pre-lockdown** | **During lockdown** | **P-value**  **(Pre- vs During Lockdown)** |
| **% time in range**  TIR: 3.9-10mmol/L (70 -180mg/dL) | 54.7 (40.8-63.6) | 60.4 (47.9-70.0) | 0.05* | 57.0 (43.6-67.4) | 59.8 (45.2-69.3) | 0.004* |
| **% time in euglycaemia**  3.9-7.8mmol/L (70 -140mg/dL) | 32.7 (25.1-39.6) | 37.7 (28.6-45.3) | 0.004* | 35.3 (23.0-43.0) | 35.6 (23.1-46.0) | 0.08 |
| **% time in hypoglycaemia**  TBR1: <3.9mmol/L (<70mg/dL)  TBR2: <3.0mmol/L (<54 mg/dL)  TBR3: <2.8mmol/L (<50mg/dL) | 2.6 (1.3-6.8)  0.4 (0.1-1.5)  0.3 (0.1-1.0) | 4.3 (1.5-7.2)  0.7 (0.3-2.1)  0.3 (0.1-1.1) | 0.14  0.66  0.83 | 4.0 (1.6-7.5)  0.9 (0.3-1.9)  0.6 (0.2-1.5) | 3.8 (1.4-6.6)  0.9 (0.3-2.1)  0.5 (0.1-1.5) | 0.02*  0.07  0.04* |
| **% time in hyperglycaemia**  TAR1: >10mmol/L (>180mg/dL)  TAR2: >13.9 mmol/L (>250mg/dL) | 39.2 (28.6-53.3)  11.9 (6.1-17.0) | 34.2 (23.6-43.9)  8.4 (4.9-16.2) | 0.07  0.34 | 37.7 (27.0-51.7)  11.3 (5.5-20.0) | 33.8 (24.0-50.9)  9.3 (4.0-19.9) | 0.06  0.009* |
| **Glycaemic variability measures**  Mean  GMI (%)  GMI (mmol/mol)  Standard deviation  CV (%)  LBGI  MAG | 9.2 (8.6-10.2)  7.3 (7.1-7.7)  56.0 (53.7-60.2)  3.6 (2.9-4.0)  36.8 (34.2-42.8)  0.7 (0.4-1.8)  2.6 (2.3-3.0) | 8.9 (8.1-9.6)  7.1 (6.8-7.4)  54.6 (50.8-57.9)  3.4 (2.8-3.9)  38.8 (33.4-41.6)  1.1 (0.5-1.6)  2.5 (2.2-2.8) | 0.06  0.06  -  0.02*  0.74  0.14  0.11 | 9.2 (8.2-10.3)  7.3 (6.8-7.7)  56.0 (51.3-61.2)  3.6 (3.1-4.1)  39.2 (34.8-42.3)  1.0 (0.5-1.8)  2.5 (2.1-3.1) | 9.0 (8.0-10.6)  7.2 (6.8-7.9)  55.1 (50.4-62.6)  3.4 (2.9-3.9)  37.6 (33.2-40.9)  0.9 (0.5-1.7)  2.5 (2.1-2.8) | 0.09  0.09  -  <0.001*  0.003*  0.05*  <0.001* |

**S1 Table**: **Pairwise analysis** of glycaemic outcomes for CSII and MDI users in **adults**. Participants used rtCGM/ isCGM for at least 70% of time with at least 70% data uploaded for both time periods (as per consensus recommendations). All data presented as median (IQR). Abbreviations: CSII, continuous subcutaneous insulin infusion; CV, coefficient of variation; GMI, glucose management indicator; LBGI, low blood glucose index; MAG, mean absolute glucose; MDI, multiple daily injections of insulin; TAR, time above range; TBR, time below range; TIR, time in range.
