# Supplementary material for: Improved glycaemia during the Covid-19 pandemic lockdown is sustained post-lockdown and during the “Eat Out to Help Out” Government Scheme, in adults with Type 1 diabetes in the United Kingdom
Source: PLoS One. 2021 Jul 20;16(7):e0254951. doi: 10.1371/journal.pone.0254951 (PMC8291633; doi:10.1371/journal.pone.0254951)
Supplement: S2 Table — Participants used rtCGM/ isCGM for at least 70% of time with at least 70% data uploaded for both time periods (as per consensus recommendations). All data presented as median (IQR). Abbreviations: CV, coefficient of variation; GMI, glucose management indicator; isCGM, intermittently scanning continuous glucose monitoring; LBGI, low blood glucose index; MAG, mean absolute glucose; rtCGM, real-time continuous glucose monitoring; TAR, time above range; TBR, time below range; TIR, time in range. (DOCX) [file pone.0254951.s002.docx]

|  | **rtCGM (n=43)** | | | **isCGM (n=78)** | | |
| --- | --- | --- | --- | --- | --- | --- |
|  | **Pre-lockdown** | **During lockdown** | **P-value**  **(Pre- vs During Lockdown)** | **Pre-lockdown** | **During lockdown** | **P-value**  **(Pre- vs During Lockdown)** |
| **% time in range**  TIR: 3.9-10mmol/L (70 -180mg/dL) | 57.6 (43.5-66.0) | 63.1 (45.5-69.6) | 0.04* | 54.6 (42.7-64.9) | 59.8 (43.9-69.3) | 0.005* |
| **% time in euglycaemia**  3.9-7.8mmol/L (70 -140mg/dL) | 33.7 (23.0-39.9) | 36.1 (22.7-45.9) | 0.04* | 33.8 (25.3-40.6) | 36.0 (27.0-44.6) | 0.02* |
| **% time in hypoglycaemia**  TBR1: <3.9mmol/L (<70mg/dL)  TBR2: <3.0mmol/L (<54 mg/dL)  TBR3: <2.8mmol/L (<50mg/dL) | 1.6 (0.7-6.5)  0.3 (0.1-1.3)  0.2 (0.1-0.9) | 3.7 (1.0-6.6)  0.8 (0.1-1.6)  0.4 (0.1-1.0) | 0.08  0.13  0.10 | 4.3 (2.7-7.9)  1.1 (0.4-2.8)  0.7 (0.2-2.3) | 4.1 (2.1-7.2)  1.0 (0.3-2.3)  0.5 (0.2-1.6) | 0.02*  0.02*  0.005* |
| **% time in hyperglycaemia**  TAR1: >10mmol/L (>180mg/dL)  TAR2: >13.9 mmol/L (>250mg/dL) | 38.8 (29.2-54.5)  11.3 (5.2-22.5) | 33.0 (22.9-50.9)  8.2 (4.5-19.3) | 0.01*  0.15 | 38.2 (27.3-48.9)  11.5 (6.1-18.2) | 34.3 (24.3-48.6)  9.2 (4.4-17.1) | 0.14  0.02* |
| **Glycaemic variability measures**  Mean  GMI (%)  GMI (mmol/mol)  Standard deviation  CV (%)  LBGI  MAG | 9.3 (8.5-10.8)  7.3 (7.0-7.9)  56.5 (52.7-63.1)  3.5 (2.8-3.8)  35.9 (32.7-41.1)  0.5 (0.2-1.6)  3.0 (2.7-3.2) | 9.0 (7.9-10.4)  7.2 (6.7-7.8)  55.1 (49.9-61.7)  3.2 (2.8-3.7)  37.7 (32.9-40.4)  0.9 (0.3-1.7)  2.8 (2.5-3.2) | 0.02*  0.02*  -  0.09  0.79  0.09  0.06 | 9.2 (8.2-10.2)  7.3 (6.8-7.7)  56.0 (51.3-60.7)  3.6 (3.2-4.2)  40.0 (35.7-43.2)  1.1 (0.7-1.9)  2.4 (2.1-2.6) | 9.0 (8.1-10.1)  7.2 (6.8-7.6)  55.1 (50.8-59.8)  3.5 (2.9-3.9)  38.1 (34.0-41.4)  1.1 (0.6-1.7)  2.2 (2.0-2.5) | 0.23  0.23  -  <0.001*  <0.001*  0.03*  0.001* |

**S2 Table**: **Pairwise analysis** of glycaemic outcomes for rtCGM and isCGM users in **adults**. Participants used rtCGM/ isCGM for at least 70% of time with at least 70% data uploaded for both time periods (as per consensus recommendations). All data presented as median (IQR). Abbreviations: CV, coefficient of variation; GMI, glucose management indicator; isCGM, intermittently scanning continuous glucose monitoring; LBGI, low blood glucose index; MAG, mean absolute glucose; rtCGM, real-time continuous glucose monitoring; TAR, time above range; TBR, time below range; TIR, time in range.
