# Supplementary material for: Improved glycaemia during the Covid-19 pandemic lockdown is sustained post-lockdown and during the “Eat Out to Help Out” Government Scheme, in adults with Type 1 diabetes in the United Kingdom
Source: PLoS One. 2021 Jul 20;16(7):e0254951. doi: 10.1371/journal.pone.0254951 (PMC8291633; doi:10.1371/journal.pone.0254951)
Supplement: S3 Table — Participants used rtCGM/ isCGM for at least 70% of time with at least 70% data uploaded for all four time periods (as per consensus recommendations). P-value calculated by Friedman test comparing glycaemic variables between the four time periods. All data presented as median (IQR). Abbreviations: CV, coefficient of variation; GMI, glucose management indicator; LBGI, low blood glucose index; MAG, mean absolute glucose; TAR, time above range; TBR, time below range; TIR, time in range. (DOCX) [file pone.0254951.s003.docx]

|  | **Pre lockdown** | **During lockdown** | **Immediately after lockdown** | **1 month after lockdown** | **P -value** |
| --- | --- | --- | --- | --- | --- |
| **% time in range**  TIR: 3.9-10mmol/L (70 -180mg/dL) | 55. 1 (43.7, 61.5) | 60.2 (44.2, 61.7) | 56.0 (45.1-60.6) | 54.0 (44.2, 57.2) | 0.97 |
| **% time in euglycaemia**  3.9-7.8mmol/L (70 -140mg/dL) | 34.4 (24.2, 42.6) | 34.7 (26.7, 41.9) | 35.3 (27.6, 41.1) | 35.2 (27.6, 37.0) | 0.72 |
| **% time in hypoglycaemia**  TBR1: <3.9mmol/L (<70mg/dL)  TBR2: <3.0mmol/L (<54 mg/dL)  TBR3: <2.8mmol/L (<50mg/dL) | 2.8 (1.3, 4.5)  0.2 (0.1, 0.8)  0.08 (0.0, 0.47) | 3.1 (1.4, 6.1)  0.4 (0.2, 1.6)  0.24 (0.15, 0.77) | 3.1 (1.9, 4.0)  0.3 (0.1, 0.8)  0.18 (0.09, 0.49) | 3.1 (1.4, 3.6)  0.2 (0.1, 0.9)  0.05 (0.04, 0.44) | 0.49  0.25  0.03* |
| **% time in hyperglycaemia**  TAR1: >10mmol/L (>180mg/dL)  TAR2: >13.9 mmol/L (>250mg/dL) | 37.9 (33.8, 54.4)  15.4 (8.6, 28.2) | 37.6 (32.4, 51.6)  16.1 (9.8, 24.1) | 39.5 (34.8, 49.6)  15.7 (10.2, 19.7) | 41.3 (38.8, 50.4)  19.0 (13.3, 21.2) | 0.94  0.68 |
| **Glycaemic variability measures**  Mean  GMI (%)  GMI (mmol/mol)  Standard deviation  CV (%)  LBGI  MAG | 9.5 (9.0, 11.2)  7.4 (7.2-8.1)  57.6 (55.1-65.4)  4.4 (3.4, 4.5)  40.7 (38.0, 45.8)  0.9 (0.4-1.1)  3.3 (3.1, 3.6) | 9. 2 (8.8, 10.8)  7.3 (7.1- 8.0)  56.0 (54.2 - 63.5)  4.1 (3.8, 4.2)  42. 6 (38.0, 43.8)  0.9 (0.4-1.4)  3.2 (3.0, 3.5) | 9. 6 (9.0, 10.5)  7.4 (7.2- 7.8)  57.8 (55.3- 61.9)  4.1 (3.6, 4.6)  41.0 (38.5, 44.9)  0.8 (0.6-1.0)  3.2 (3.0, 3.5) | 9.7 (9.0, 10.5)  7.5 (7.2- 7.8)  58.5 (55.3 - 62.1)  4.3 (3.7, 4.7)  41. 4 (37.9, 46.7)  0.9 (0.5-0.9)  3.1 (3.0, 3.5) | 0.60  0.60  -  0.29  0.90  0.52  0.20 |

**S3 Table: Paediatric analysis** of glycaemic outcomes pre-, during, immediately after and a month after lockdown (n=13). Participants used rtCGM/ isCGM for at least 70% of time with at least 70% data uploaded for all four time periods (as per consensus recommendations). P-value calculated by Friedman test comparing glycaemic variables between the four time periods. All data presented as median (IQR). Abbreviations: CV, coefficient of variation; GMI, glucose management indicator; LBGI, low blood glucose index; MAG, mean absolute glucose; TAR, time above range; TBR, time below range; TIR, time in range.
