# Supplementary material for: Improved glycaemia during the Covid-19 pandemic lockdown is sustained post-lockdown and during the “Eat Out to Help Out” Government Scheme, in adults with Type 1 diabetes in the United Kingdom
Source: PLoS One. 2021 Jul 20;16(7):e0254951. doi: 10.1371/journal.pone.0254951 (PMC8291633; doi:10.1371/journal.pone.0254951)
Supplement: S1 Fig — Abbreviations: FSL, flash glucose monitoring; rtCGM, real-time continuous glucose monitoring. (DOCX) [file pone.0254951.s004.docx]

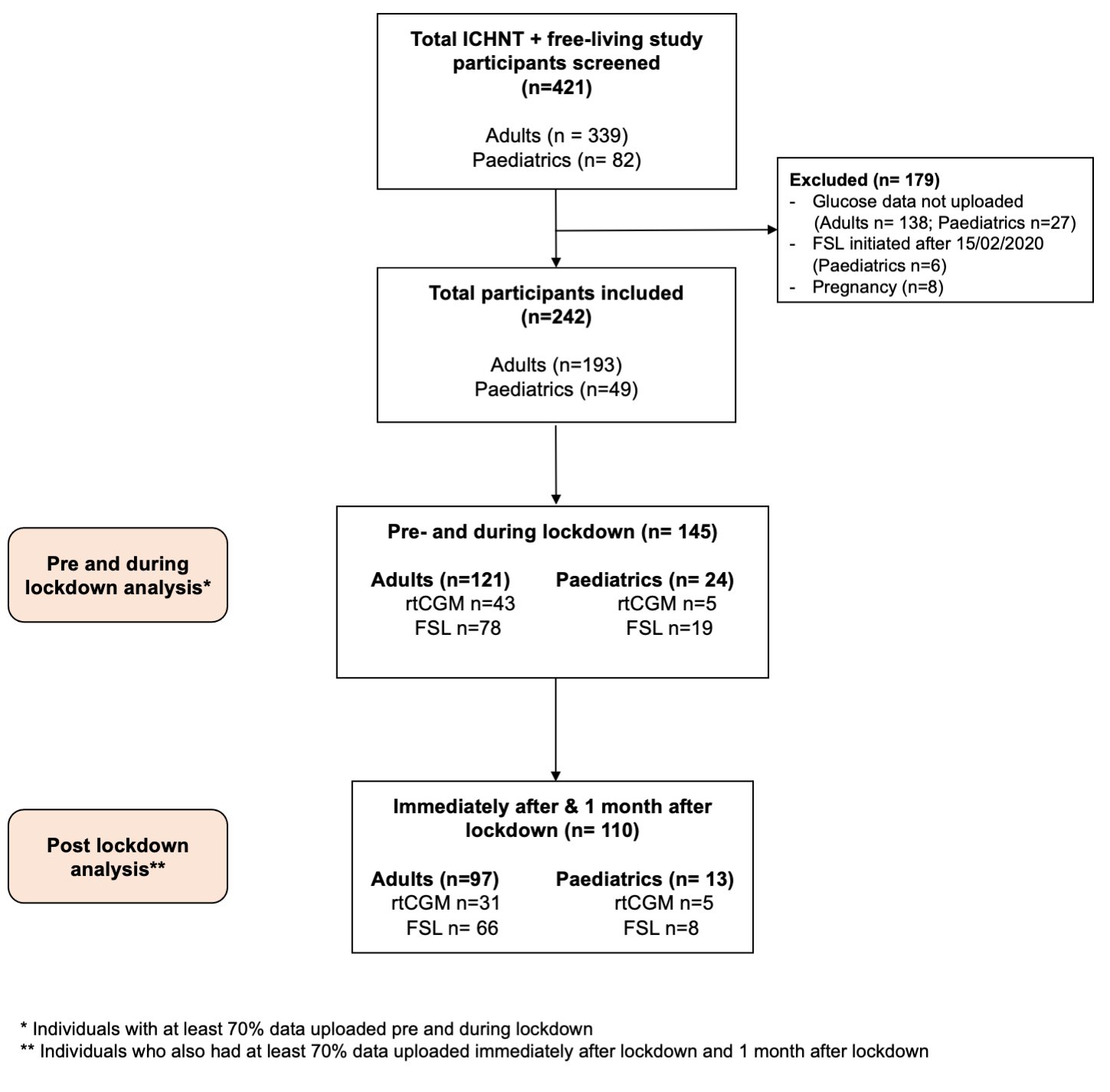


**S1 Fig:** Recruitment flowchart. Abbreviations: FSL, flash glucose monitoring; rtCGM, real-time continuous glucose monitoring
