# Supplementary material for: Improved glycaemia during the Covid-19 pandemic lockdown is sustained post-lockdown and during the “Eat Out to Help Out” Government Scheme, in adults with Type 1 diabetes in the United Kingdom
Source: PLoS One. 2021 Jul 20;16(7):e0254951. doi: 10.1371/journal.pone.0254951 (PMC8291633; doi:10.1371/journal.pone.0254951)
Supplement: S2 Fig — The “most deprived” tertile include deciles 1–3 (n = 52), the “moderately deprived” tertile includes deciles 4–5 (n = 43) and the “least deprived” tertiles includes deciles 6–10 (n = 50). Abbreviations: TIR, time in range. (DOCX) [file pone.0254951.s005.docx]

**
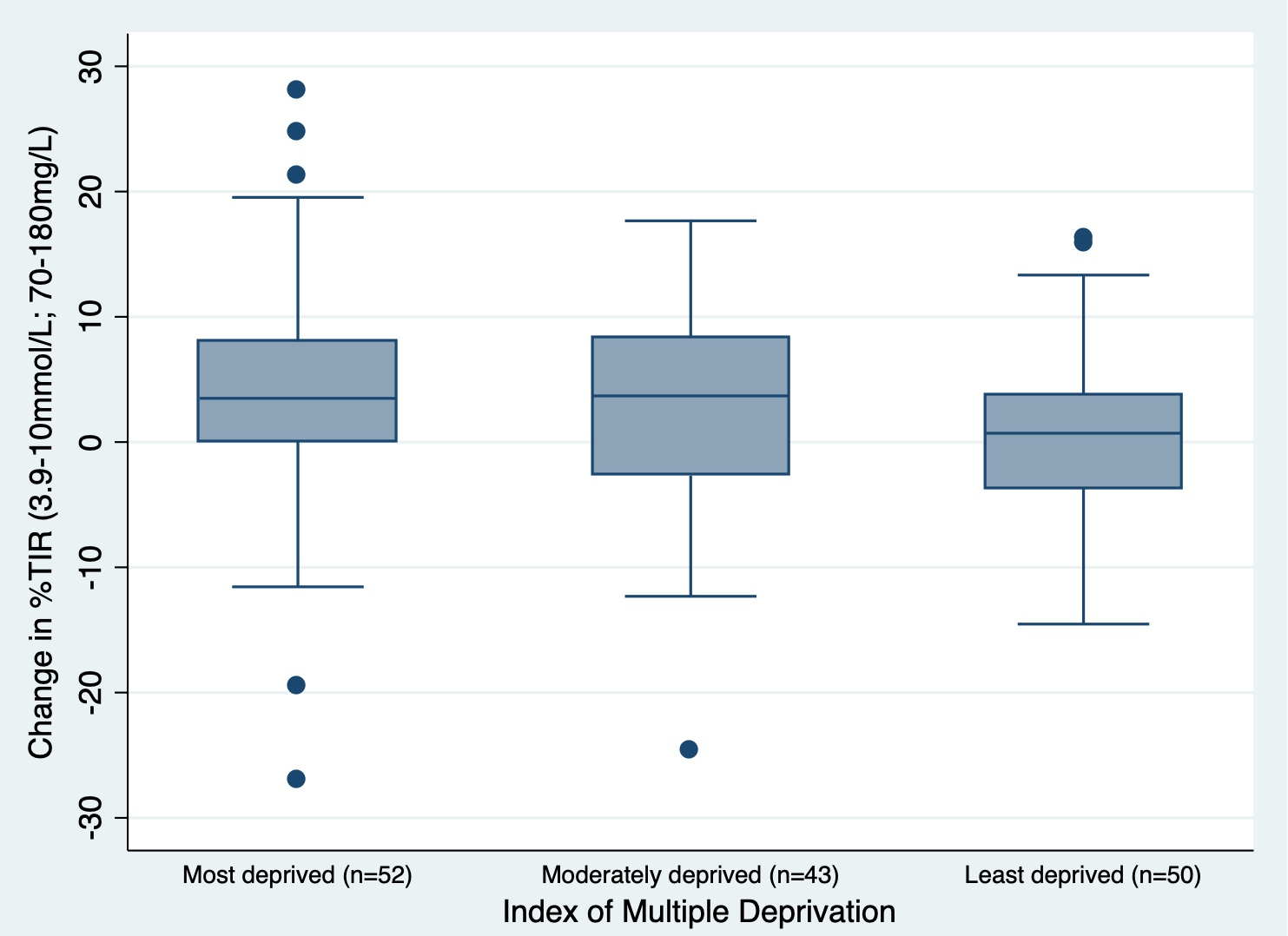
**

**S2 Fig:** Box plot to demonstrate the change in %TIR by tertiles for adults and children included in the pair-wise analysis (n=145) using the English Index of Multiple Deprivation 2019. The “most deprived” tertile include deciles 1-3 (n=52), the “moderately deprived” tertile includes deciles 4-5 (n=43) and the “least deprived” tertiles includes deciles 6-10 (n=50). Abbreviations: TIR, time in range.
